# Clinical Features and Outcomes of Patients With Chronic Myeloid Leukemia Presenting With Isolated Thrombocytosis: A Systematic Review and a Case From Our Institution

**DOI:** 10.7759/cureus.8788

**Published:** 2020-06-23

**Authors:** Dawood Findakly, Waqas Arslan

**Affiliations:** 1 Internal Medicine, Creighton University Arizona Health Education Alliance/Valleywise Health Medical Center, Phoenix, USA; 2 Hematology and Oncology, Creighton University Arizona Health Education Alliance/Valleywise Health Medical Center, Phoenix, USA; 3 Hematology and Oncology, Creighton University Maricopa Medical Center, Phoenix, USA

**Keywords:** isolated thrombocytosis, bcr-abl positive, chronic myeloid leukemia, systematic review

## Abstract

Chronic myeloid leukemia (CML) represents a common condition in the spectrum of myeloproliferative disorders (MPD). It classically exhibits leukocytosis, but rarely presents with isolated thrombocytosis. This paper is designed to review the clinicopathologic features, treatment, and outcomes of patients with CML who present with isolated thrombocytosis.

We searched PubMed, MEDLINE®, ScienceDirect, and Scopus for English-language articles about case series and case reports for the period 2000-2020 with the terms “chronic myeloid leukemia” and “thrombocytosis” and pooled them with a case from our institution. Cases were also incorporated from the reference list and screened for inclusion. A total of 20 cases were included in the final cohort. The male-to-female ratio was 1:1.86. The mean age of the patients at the time of initial diagnosis was 40.5 years (range: 9-77 years). Out of 17 cases with available data, seven (41%) were asymptomatic and found to have thrombocytosis incidentally upon routine blood work. Five cases (29.4%) either had a history of thrombotic events or presented with severe thrombotic complications, including ischemic cerebrovascular accidents (CVA), myocardial infarction (MI), pulmonary embolism (PE), and/or miscarriages. Four cases (23.5%) had more than one symptom at presentation, including headache, syncope, and bruising. The average platelet count was 1,923 × 10^9^/L (range: 584-8,688 × 10^9^/L), and one case (5%) had anemia. The bone marrow (BM) examination showed normal cellularity and normal myeloid to erythroid (M/E) ratio in seven (50%) and 11 (84.6%) out of the 14 and 13 cases with reported data, respectively. Moreover, megakaryocytes in the BM were small in 10 cases (71.4%), pleomorphic in three cases (21.4%), and dysplastic in one case (7.1%).

Accurate differentiation among MPD subtypes and the exclusion of CML is critical in reaching a proper diagnosis to decide on proper therapy and eventually modify outcomes. Prompt evaluation for the precise diagnosis of patients presenting with isolated marked thrombocytosis will help expedite their diagnosis and initiation of a specific tyrosine kinase inhibitor (TKI) therapy, thereby promptly inducing remission, preventing thrombotic complications, and avoiding adverse drug events, which would eventually improve outcomes.

## Introduction and background

Chronic myeloid leukemia (CML) belongs to the spectrum of myeloproliferative disorders (MPD), which are myeloid lineage clonal disorders that also include essential thrombocythemia, polycythemia vera, and myelofibrosis [1]. It accounts for 15-20% of all adult leukemia cases, with an estimated incidence of fewer than 5,000 new cases in the United States per year [2,3]. CML is characterized by the excess of white blood cells (WBCs) resulting from the uncontrolled proliferation of mature granulocytes and their precursors [3]. When patients present with isolated thrombocytosis, testing for the Philadelphia chromosome or BCR-ABL is essential to identify CML cases among them [4]. This study reviews the clinical characteristics, diagnosis, therapeutic modalities, and prognosis regarding patients with isolated thrombocytosis as an initial presentation of CML.

## Review

Aim of the study

This review aimed to investigate the available published data on the clinical characteristics, treatment, and prognosis of CML in patients presenting with isolated thrombocytosis.

Patients and methods

Literature Search Strategy

**Figure 1:**
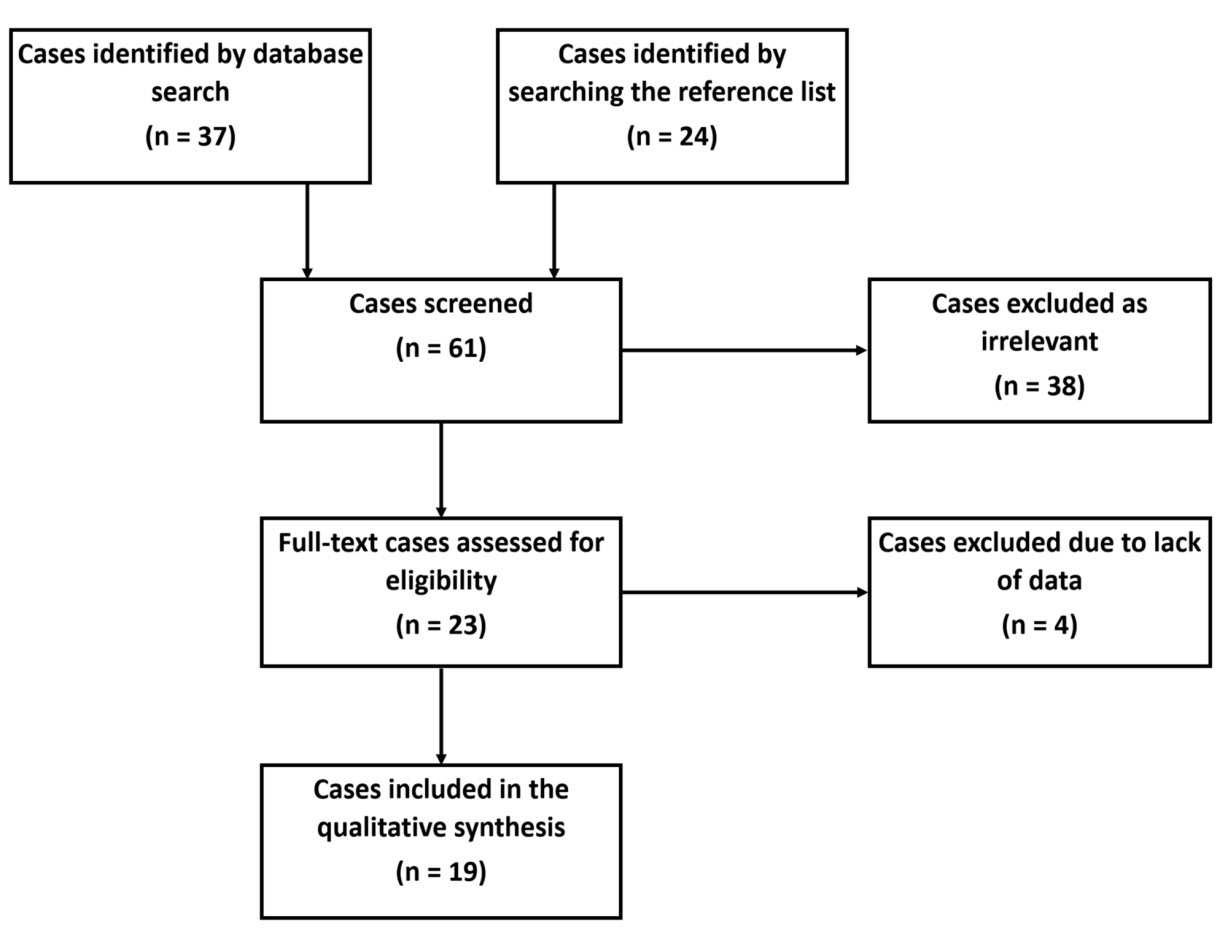
The PRISMA flow diagram detailing the cases of CML that presented with isolated thrombocytosis PRISMA: Preferred Reporting Items for Systematic Reviews and Meta-Analyses; CML: chronic myeloid leukemia

We performed a systematic review of the PubMed, MEDLINE®, ScienceDirect, and Scopus databases for the published literature in the English language during the period 2000-2020 that reported isolated thrombocytosis as an initial presentation in laboratory-confirmed CML patients. The recognized list of cases and abstracts were evaluated for any additional articles of interest from reference lists and pooled with a case from our institution (Figure 1).

Data Extraction and Statistical Analysis

We extracted data, when available, on age at the time of diagnosis, peripheral blood (PB) and bone marrow (BM) examination findings, splenic enlargement, diagnosis, treatment, complications, duration of follow-ups, and outcomes. One case from our institution was also included in our analysis. The pooled data were interpreted and summarized through descriptive statistics using central tendency and dispersion measures.

Results

**Figure 2:**
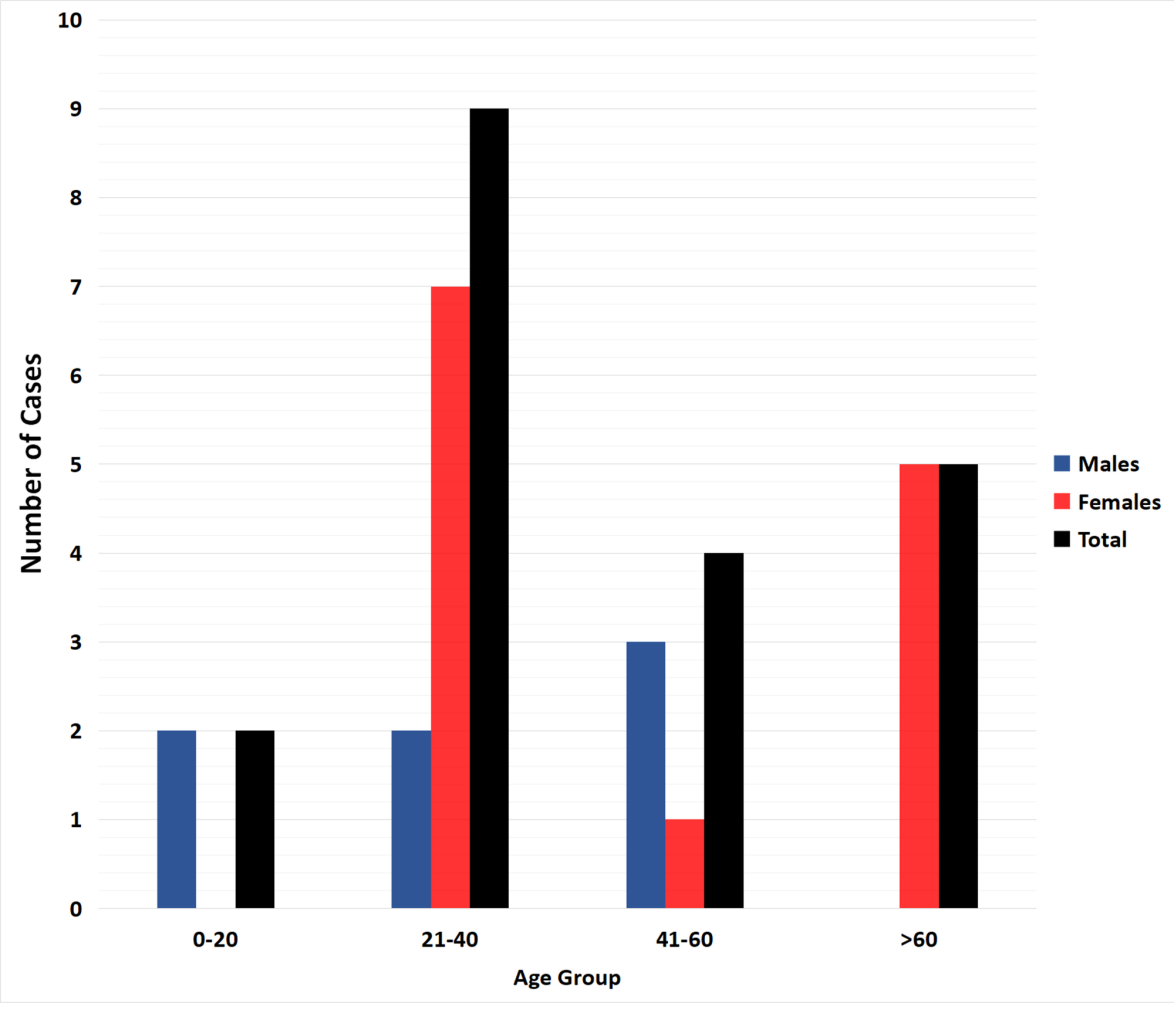
Age and gender distribution of CML patients presenting with isolated thrombocytosis The bar graph represents the number of patients with CML for the specific age and gender group CML: chronic myeloid leukemia

We recognized 19 published studies where CML diagnosis was established in the setting of isolated thrombocytosis. Furthermore, we included one case from our institutional database. The final cohort consisted of a total of 20 patients with CML who presented with isolated thrombocytosis. The demographics, clinical features, and outcomes of the 20 cases are summarized in Table 1 [5-19]. The mean age at diagnosis was 40.5 years (range: 9-77 years), with 15 (75%) being 60 years of age or younger at the time of initial diagnosis. The final data consisted of seven males and 13 females (male-to-female ratio: 1:1.86). Age and gender distribution of patients are summarized in Figure 2.

**Table 1:** Summary of the available publications (including our case) regarding CML patients who present with isolated thrombocytosis M: male; F: female; HA: headache; NM: not mentioned; CVA: cerebrovascular accident; PE: pulmonary embolism; h/o: history of; MI: myocardial infarction; WNL: within normal limits; PLT: platelet count; Hgb: hemoglobin; WBC: white blood cells; BM: bone marrow; M:E ratio: myeloid-to-erythroid ratio; IFNA2b: interferon-alpha 2b; HU: hydroxyurea; Ara-c: cytosine arabinoside; PLTP: plateletpheresis; SCT: stem cell transplant; R: refractory; intol.: intolerance; TKI: tyrosine kinase inhibitor; peg-IFNa-2a: pegylated-interferon alpha-2a; CML: chronic myeloid leukemia; CAD: coronary artery disease; FU: follow-up; APD: alive, persistent disease; AIN: alive, in remission; DOD: died of disease; GVHD: graft-versus-host disease; DUR: died of unknown reason

Author	Year	Age (years)	Gender	Symptoms	PLT (x109/L)	Hgb (g/dl)	WBC (x109/l)	BM	Splenomegaly	Treatment	Complication	Duration of FU (months)	Outcome
Fadila et al. [5]	2000	37	M	HA	2,520	13.2	13.5	Hypercellularity, pleomorphic megakaryocytic, normal M:E ratio	Present	IFNA2b, HU, Ara-c	CML blast phase	32	APD
Damaj, et al. [6]	2002	28	F	NM	1,723	13.3	12.9	NM	NM	HU	NM	72	AIN
Michiels et al. [7]	2003	65	F	Asymptomatic	584	12.4	4.3	Normal cellularity, small megakaryocytes, normal M:E ratio	Absent	HU	NM	144	AIN
Girodon et al. [8]	2005	52	M	Asymptomatic, blood donor	672	12.9	7.9	Hypercellularity, small megakaryocytes, normal M:E ratio	Absent	Imatinib	NM	7	AIN
Rice et al. [9]	2005	27	F	Miscarriage, CVA, PE	1,880	14	8.4	NM	Absent	Anagrelide, HU, imatinib, PLTP, SCT	CVA	48	APD
Rice et al. [9]	2005	42	F	Asymptomatic	700	WNL	WNL	NM	Absent	Anagrelide, HU, imatinib, steroids, SCT	CAD	36	DOD (GVHD)
Breccia et al. [10]	2008	43	M	Asymptomatic	1,310	14	2.8	Normal cellularity, dysplastic megakaryocytes, M:E ratio, NM	NM	HU (R), then switched to imatinib	Leukopenia	15	AIN
Niekerk et al. [11]	2012	68	F	Asymptomatic, h/o follicular lymphoma	2,062	WNL	WNL	Hypercellularity, small megakaryocytes, high M:E ratio	NM	TKI (intol.), then switched to peg-IFNa-2a	TKI intol.: acrocyanosis, dyspnea	9	AIN
Byun et al. [4]	2014	21	F	Abdominal pain (ruptured corpus luteal cyst)	3,777	10.1	10	Hypercellularity, small megakaryocytes, high M:E ratio	Absent	HU (R), then switched to imatinib	NM	3	AIN
Ebrahem et al. [12]	2015	39	F	Syncope, seizure, MI	2,500	WNL	13.8	NM	Present	Dasatinib, PLTP	NM	NM	AIN
Yilmaz et al. [13]	2016	77	F	Asymptomatic, h/o CVA	711	13.8	5.2	Normal cellularity, small megakaryocytes, normal M:E ratio	Absent	Imatinib	NM	3	AIN
Yilmaz et al. [13]	2016	70	F	Asymptomatic, h/o CVA, MI	1,853	13	9.2	Normal cellularity, small megakaryocytes, normal M:E ratio	Absent	Imatinib	NM	12	AIN
Yilmaz et al. [13]	2016	30	M	Flu-like	1,277	15.7	11.1	Hypercellularity, small megakaryocytes, normal M:E ratio	Absent	Imatinib (R), then switched to nilotinib (R), then switched to dasatinib (R), pending SCT	NM	12	APD
Boklan et al. [14]	2017	10	M	Bruising	8,688	11.2	WNL	Hypercellularity, pleomorphic megakaryocytic, normal M:E ratio	Absent	PLTP, HU, imatinib (intol.), then switched to dasatinib	TKI intol.: acrocyanosis, dyspnea	5	AIN
Huho et al. [15]	2018	9	M	Cough, fatigue, HA, bruising	2,500	13	8	Normal cellularity, small megakaryocytes, normal M:E ratio	Absent	Dasatinib	NM	9	AIN
Soliman et al. [16]	2019	46	M	Chest pain, dyspnea	1,065	11.7	6.7	Normal cellularity, small megakaryocytes, normal M:E ratio	Absent	Dasatinib	NM	24	AIN
Aslan et al. [17]	2019	25	F	NM	678	WNL	WNL	NM	NM	Imatinib	Leukopenia	3	AIN
Aslan et al. [17]	2019	40	F	NM	747	WNL	8.8	NM	NM	Imatinib	NM	3	AIN
Han et al. [18]	2020	61	F	Liver mass (adenocarcinoma)	2,464	11.1	7	Hypercellularity, pleomorphic megakaryocytic, normal M:E ratio	Absent	HU, imatinib	NM	6.5	DUR
Findakly and Arslan [19] (our case)	2020	21	F	Syncope	764	13.3	7.2	Normal cellularity, small megakaryocytes, normal M:E ratio	Absent	Dasatinib, pending SCT	NM	32	AIN

Among patients with reported data, all (100%) reported being tested negative for the Janus kinase 2 (JAK2), calreticulin (CALR), and thrombopoietin receptor (MPL) gene mutations. Moreover, all (100%) tested positive for BCR-ABL translocation and, therefore, was diagnosed with chronic-phase CML. Moreover, out of the 15 patients with available data describing splenomegaly, either upon physical examination or imaging, only two (13.3%) had splenomegaly, while it was absent in the 13 patients (86.7%). The average platelet count was 1,923 × 10^9^/L (range; 584-8,688 × 10^9^/L). Only one patient (5%) reported anemia with a hemoglobin level of 10.1 g/dl. The patients' characteristics are summarized in Table 2.

**Table 2:** Patient characteristics n: number; M: male; F: female; PLT: platelet count; WBC: white blood cells; Hgb: hemoglobin

Patient characteristics	Value
Patients (n)	20
Gender (M/F)	7/13
Age (years), mean (range)	40.5 (9-77)
PLT (x10^9^/L), mean (range)	1,923 (584-8,688)
WBC count (x10^9^/L), mean (range)	8.2 (2.8-13.89)
Hgb (g/dl), mean (range)	12.8 (10.1-15.7)
Splenomegaly (n, %)	2, 13.3%

Data on BM characteristics were reported for 14 patients. Interestingly, a peculiarly normal cellularity and myeloid-to-erythroid (M:E) ratio was reported in seven (50%) and 11 (84.6%) out of the 14 and 13 patients with reported data, respectively. Moreover, megakaryocytes were small in 10 patients (71.4%), pleomorphic in three patients (21.4%), and dysplastic in a single patient (7.1%), respectively. Nineteen patients (95%) were in a chronic CML phase. The case from our institution tested negative for BCR‐ABL gene mutation in PB, but subsequent BM aspiration and biopsy performed later were consistent with CML.

In terms of treatment modalities, hydroxyurea (HU) was used as part of initial therapy in nine patients (45%), out of which four (44%) received it solely; two (22%) were noticed to have a refractory thrombocytosis and, therefore, were switched to tyrosine kinase inhibitor (TKI) therapy. TKI therapy was used in 17 patients (85%), out of which nine patients (53%) received it solely. Fifteen patients (88%) were treated with a single TKI, one patient (6%) was treated with two TKIs, and another patient (6%) was treated with more than two TKIs given refractory disease. The initial TKI therapy was imatinib and dasatinib in 12 (75%) and four (25%) of the 16 cases that specifically reported the name of the TKI medication used. Furthermore, three patients (15%) received plateletpheresis as part of the treatment, and four patients (25%) were enrolled for stem cell transplantation (SCT).

The mean duration of follow-up was 25 months (range 3-144 months). Complications during therapy were reported in seven cases (35%), where one case (5%) progressed to the blastic-phase CML, two female patients (10%) developed significant thrombotic complications despite being on HU, two (10%) developed leukopenia, and two (10%) had TKI-intolerance out of which one reported to develop acrocyanosis and dyspnea. Notably, thrombotic complications occurred after diagnosis with two female patients (100%) being treated with HU preceding imatinib therapy. Furthermore, five (38.4%) out of the 13 female patients had previously had and/or developed major thrombotic events, including cerebrovascular accident (CVA), myocardial infarction (MI), coronary artery disease (CAD), pulmonary embolism (PE), and miscarriage; those patients had a mean age of 51 years (range: 27-77 years).

In terms of outcomes, 15 patients (75%) were alive and in remission at the time of follow-up, three patients (15%) were alive with persistent disease, and two had died (10%). Both (100%) deaths had occurred during the chronic CML phase; the first patient (5%) had died of a complication of the SCT where the patient had developed graft versus host disease (GVHD), and the second patient (5%) had died of an obscure reason. Moreover, both had been females with a mean age of 51.5 years (range: 42-61 years), and death had occurred at a mean duration of follow-up of 21 months (range: 6.5-36 months) (Table 3).

**Table 3:** Summary of patient outcomes N: number; AIR: alive, in remission; APD: alive, with persistent disease

Patient outcomes	N, %
AIR	15, 75%
APD	3, 15%
Deceased	2, 10%

Discussion

In our cohort, several findings are noteworthy. First, Upon PB examination, the average platelet count was 1,923 × 10^9^/L (range: 584-8,688 × 10^9^/L). Second, unlike classical CML, splenomegaly was present in only about 13%, while normal BM cellularity and M:E ratio were noted in 50% and approximately 85% of patients with reported data, respectively. Third, female patients had worse morbidity and mortality rates compared to male patients. Nearly 38% of the female patients had previously had and/or developed major thrombotic events with a mean age of 51 years (range: 27-77 years).

Typical PB smear features of CML include marked leukocytosis due to neutrophils being in all maturation stages, with a WBC count ranging between 12-1,000 × 10^9^/L [20]. The typical platelet count at diagnosis in CML could vary from normal range to less than 1,000 × 10^9^/L [1]. Although thrombocytosis is a relatively common presenting feature, it rarely exceeds 1,000 × 10^9^/L. Moreover, the typical BM aspirate findings for patients with CML include hyperplasia of granulocytic progenitor cells with markedly elevated M:E ratio contributing to hypercellularity of BM. Other non-specific BM findings constitute an increase in vascularity and reticulin fibrosis [21]. Moreover, the number and size of erythroid lineage are decreased, and the megakaryocytes are distinguished by being smaller than normal with nuclei that are characteristically hypolobulated [22].

Regarding modalities of therapy, HU is an antimetabolite that acts by reducing the platelet count in patients with essential thrombocythemia and also aids in preventing thrombosis. Moreover, imatinib, nilotinib, and dasatinib were reported as treatment modalities in cases included in this review. Of those, nilotinib and dasatinib are second-generation TKI medications with superior efficacy and higher potency compared to imatinib as a first-line treatment for the chronic-phase CML and cases with resistance or intolerance to prior TKI therapy [23,24]. False-negative results may occur due to poor quality or failure to promptly stabilize RNA sample at the time of sample collection, leading to a failure of the polymerase chain reaction (PCR), atypical transcripts, or during therapeutic monitoring [25-27].

The initial PB fluorescence in-situ hybridization (FISH) testing for the patient we included from our institution was negative for the BCR‐ABL gene mutation. However, consequent BM aspiration and biopsy performed as platelet count continued to rise was consistent with CML. Therefore, we underscore the importance of providers routinely pursuing genetic testing for the BCR/ABL gene in both PB and BM as part of the workup for patients with isolated thrombocytosis to avoid misdiagnosis or delayed diagnosis of CML in this patient population.

## Conclusions

Our study illustrates that CML is a vital differential that should not be missed, particularly among patients with isolated marked thrombocytosis. Pursuing genetic testing for the BCR/ABL fusion gene in both PB and BM is essential in the diagnosis. Familiarity with this entity will help to promptly identify it, expedite proper diagnosis, and subsequently initiate specific TKI therapy that will help induce remission, prevent thrombotic complications, and avoid adverse drug events.
